# Job Exposure Matrix for Electric Shock Risks with Their Uncertainties

**DOI:** 10.3390/ijerph120403889

**Published:** 2015-04-08

**Authors:** Ximena P. Vergara, Heidi J. Fischer, Michael Yost, Michael Silva, David A. Lombardi, Leeka Kheifets

**Affiliations:** 1 Electric Power Research Institute, Environment Sector, Palo Alto, CA 94304, USA; 2 UCLA Fielding School of Public Health, Department of Biostatistics, Los Angeles, CA 90024, USA; E-Mail: Heidi.j.fischer@gmail.com; 3 Department of Environmental and Occupational Health Sciences, University of Washington School of Public Health, Seattle, WA 98195, USA; E-Mail: airion@uw.edu; 4 Enertech Consultants, Campbell, CA 95008, USA; E-Mail: msilva@enertech.net; 5 Center for Injury Epidemiology, Liberty Mutual Research Institute for Safety, Hopkinton, MA 07418, USA; E-Mail: David.Lombardi@libertymutual.com; 6 UCLA Fielding School of Public Health, Department of Epidemiology, Los Angeles, CA 90024, USA; E-Mail: kheifets@ucla.edu

**Keywords:** EMF, electric shocks, job exposure matrix, expert elicitation, uncertainty

## Abstract

We present an update to an electric shock job exposure matrix (JEM) that assigned ordinal electric shocks exposure for 501 occupational titles based on electric shocks and electrocutions from two available data sources and expert judgment. Using formal expert elicitation and starting with data on electric injury, we arrive at a consensus-based JEM. In our new JEM, we quantify exposures by adding three new dimensions: (1) the elicited median proportion; (2) the elicited 25th percentile; and (3) and the elicited 75th percentile of those experiencing occupational electric shocks in a working lifetime. We construct the relative interquartile range (rIQR) based on uncertainty interval and the median. Finally, we describe overall results, highlight examples demonstrating the impact of cut point selection on exposure assignment, and evaluate potential impacts of such selection on epidemiologic studies of the electric work environment. In conclusion, novel methods allowed for consistent exposure estimates that move from qualitative to quantitative measures in this population-based JEM. Overlapping ranges of median exposure in various categories reflect our limited knowledge about this exposure.

## 1. Introduction

Electric shocks, electric fields and magnetic fields are all part of the “electric” work environment. Electric occupations have been consistently associated with amyotrophic lateral sclerosis (ALS) [1,2]. Because the association with measured extremely low frequency magnetic fields levels is weaker [2], exposure to electric shocks has been suggested as an explanation for the observed association between electric occupations and ALS [3,4]. Thus, separation of highly correlated electric exposures remains an important public health priority [1]. An electric shock job exposure matrix (JEM) was created to provide estimates of exposure for evaluating relationships between occupational electric shocks, magnetic fields and health outcomes, in particular ALS [5]. To allow for proper inferences from any epidemiologic study, analyses should include uncertainties in exposure assignment to assess exposure misclassification.

We previously reported the development of a population-based electric shock JEM containing ordinal exposures [5]. As we expect exposure misclassification to be a significant source of bias when this JEM is applied to epidemiologic data, quantitatively assessing impacts of exposure misclassification through bias analysis is paramount for decision-making and guidance on future research [6,7].

This paper presents a description of an expanded electric shock JEM, which contains exposures quantified on a continuous scale and exposure uncertainty expressed as probability distributions using parameters determined through formal expert elicitation. We summarize the data by thirteen major occupational groups and by occupational titles, which we refer to as “occupations”. We examine how cut point decisions impact assignment of occupations into various categories. The new electric shock JEM, developed independently of magnetic fields, has potential to separate these exposures in epidemiologic analyses and highlight particular occupations for which the exposure assignment is less certain.

## 2. Experimental Section

### 2.1. National Occupational Electric Shock Injury and Fatality Data

Previously, we estimated electric shock/electrocution proportions for each of 1990 U.S. Bureau of Census (BOC-90) occupations. The BOC is a coding system devised by the U.S. Bureau of Labor Statistics to classify reported jobs. The scheme is also used for several time periods of U.S. mortality data, to which the electric shock JEM will be applied. The BOC-90 system has 501 separate categories (3-digit level) of occupation arranged into 6 summary and 13 major occupational groups. We constructed proportions using 1992–1999 U.S. Bureau of Labor Statistics injury and fatality data from the U.S. Occupational Safety and Health Administration Integrated Management Information System. Sum of injury and fatality served as the numerator, while the denominator was constructed using Current Population Survey, a monthly survey of households conducted for the U.S. Bureau of Labor Statistics [8]. National injury/fatality, data-only proportions representing fatal and non-fatal electric shocks were estimated separately by each BOC-90 occupation (the number of fatal plus non-fatal injuries per number of people for that specific occupation in the U.S.). The highest injury and fatality proportions were 8.6 and 4.2 per 10,000 workers, respectively. As previously described, experts assigned each of the BOC-90 occupations to an electric shock exposure (low, medium or high) by consensus after considering several factors for each job: energy sources, workplace safeguards, environment, worker training, protective measures, and calculated national injury/fatality proportions [5].

### 2.2. Formal Expert Elicitation

In this new effort, we assembled a panel of three experts with direct relevant, practical experience and diverse backgrounds to elicit the median proportions from zero to one and 25th/75th percentiles of uncertainty. The elicited median proportion is the probability of a worker encountering electric shocks within a working lifetime. We were interested in electric shocks ranging from those barely perceptible to noticeable to annoying, as well as painful. One assumption our experts agreed was that the frequency of perceptible shocks were correlated with electric injury, on which the elicitation was based. The experts were: an academic industrial hygienist (MY), an electrical engineer with electric utility experience and research into magnetic fields and shocks (MS) and an injury epidemiologist, with background in electrical injury worker insurance claims (DL). Experts were trained to quantify the electric-shock JEM using a formalized elicitation process described elsewhere and summarized below [9].

The purpose of the electric shock elicitation was to construct probability distributions, which represent an aggregate of each expert’s epistemic uncertainty. Formal elicitation was used as means to reduce heuristics and biases. As such, several training and follow-up conference calls and a two-day in-person meeting were used to elicit the JEM dimensions [9]. Briefly, experts were sent background peer-review literature, the U.S. Bureau of Census Occupation code manual, a description of the elicitation exercises and tasks, and contextual causes of bias. During training, the experts arrived at an electric shock working definition. Originally, experts were asked to provide 5%–95% uncertainty intervals. However, while on these calls, experts agreed they would be able to provide better estimates of 25%–75% uncertainty intervals. After several pre-training calls, exercises were given to assign median exposure proportions, intervals/quantiles, and rationale for the assignment. In these exercises, experts were encouraged to examine job factors for each occupation, rather than relying solely on national injury/fatality, data-only proportions. Independently, the experts assigned electric shock exposure proportions after the training was complete. During the in-person meeting, an iterative process was used in which experts compared elicited median proportions for a given occupation, derived from both expert judgment and data models generated prior to the meeting, to medians from similar occupations. The experts discussed and modified their exposure median proportions and arrived at a consensus [9]. The product of the elicitation exercise was three new dimensions to the JEM: (1) the elicited median proportion between zero and one; (2) the 25th percentile; and (3) the 75th percentile of this exposure of those experiencing electric shocks in a working lifetime for each of the 501 BOC-90 occupations.

### 2.3. Post-Elicitation Analyses

To provide an indicator of exposure uncertainty, the relative interquartile range (rIQR) was calculated as:

$$\mathrm{rIQR}=\frac{75th\;\%-25th\;\%}{Median}$$

We present rIQR as means within occupation groups and by occupations. We also focus on rIQR for select occupations classified as the highest exposure group in the original JEM. Note that, as rIQR depend on both the point estimate and the range of exposure, they were used only to examine relative ranking of uncertainty.

Since we were interested in the electric work environment, we also extracted geometric magnetic fields mean data from a previously published population-based magnetic fields JEM [10]. The Bowman *et al.* [10] JEM is the most complete for use in the U.S. and is coded to BOC-80 occupations (codes similar to BOC-90). No magnetic fields level data for 78 BOC-80 occupations were available. After converting the remaining 423 occupations to BOC-90, we categorized them by geometric mean magnetic fields as follows: high (≥0.30 µT), medium (0.10 µT−0.30 µT) and low (≤0.10 µT), as has typically been done in many other epidemiological studies [11,12].

Median proportions can be used in analyses as continuous or as categorized variables (e.g. high, medium, and low exposure), based on different cut points. An electric shock JEM based on continuous exposure measures is advantageous as several cut points may be examined prior to a full-scale epidemiologic study. Since there is no biological basis upon which to categorize a particular occupation, for electric shocks or magnetic fields, cut points are usually set based on exposure distribution percentiles of the cohort or controls in a case-control study. For this descriptive analysis, we divided occupations into high, medium and low exposures, using the same cut points we used in the original JEM to examine changes in exposure categorization. Cut points were defined using the distribution of elicited median electric shocks exposure proportions, created by ranking median exposure proportions across all 501 occupations.

To examine changes in the shocks and magnetic fields exposure contrasts, we selected the original cut point scheme of 67% distribution/33% derived from the injury/fatality proportion distributions used in the first electric shock JEM publication [5]. Other exposure distribution cut points have been used for the European electric shock JEM, such as 90%/75% [13]. However, to ease discussion for the comparison between the original and expanded JEM, we used only tertile cut points (67%: 0.12, 33%: 0.004906). For the expanded JEM, we also highlight the frequency of occupations from the original JEM, which lie between different median exposure ranges. We summarize the number of occupations within each of the discordant exposure cells, as these contrasts are critical for the separation of electric shocks and magnetic fields exposures in the electric work environment.

## 3. Results

We present the median proportions of workers experiencing electric shocks and rIQR elicited from the expert panel by major occupational group, then by occupation. We highlight differences between the previous electric shock JEM and expanded electric shock JEM. Last, we present the frequency of occupations obtained in the exposure contrasts for electric shocks and magnetic fields.

### 3.1. Median Exposure Proportions

#### 3.1.1. By Thirteen Major Occupation Groups 

**Figure 1:**
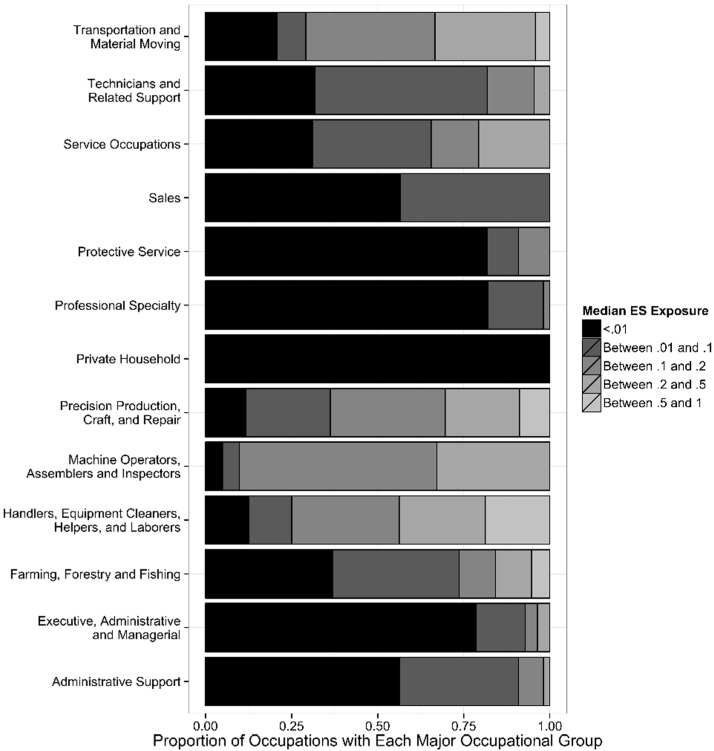
Distribution of median electric shock exposure by major occupation group.

Of the thirteen major occupation groups, the group with the most frequent medians greater than 0.50 was the precision, production, craft, and repair occupations. The highest percentage of occupations having some shocks (*i.e.*, median exposure proportions greater than 0.01—essentially the lowest median) was among machine operators, assemblers, and inspectors (95%), precision, production, craft, and repair occupations (88%) and handlers, equipment cleaners, helpers and laborers (88%) (Figure 1). Whereas groups having the lowest percentage of occupations with medians greater than 0.01 were among protective service occupations (18%), executive, administrative, and managerial occupations (21%), professional specialty occupations (18%), and private household occupations (0%).

#### 3.1.2. By Occupation

**Figure 2:**
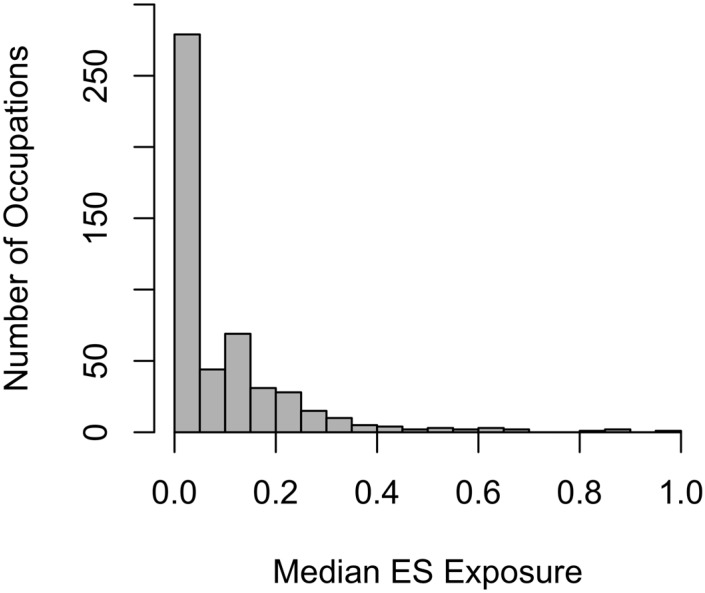
Overall distribution of median exposure for all occupations.

Approximately 42% of the 501 occupations were considered to have a low electric shock exposure and were below or equal to the electric shocks median exposure proportion of 0.01 (Figure 2). Overall, the average of the median electric shocks exposure proportions for the 501 occupations was 0.10 (Range: 0.0004906–0.97), with a positively skewed exposure distribution. Exposure proportions equaled or exceeded 0.70 for five occupations and 37 occupations fell between the exposure proportion range of 0.30–0.69 (Figure 2). The majority of occupations (64%) fell into an exposure proportion range between 0.0004906 and 0.10.

### 3.2. Uncertainty: Relative Interquartile Ranges 

#### 3.2.1. By Thirteen Major Occupation Groups

By major occupation group, the largest rIQR on average was observed for private household occupations (mean rIQR = 20.4), followed by professional specialty occupations (mean rIQR = 15.9), protective service occupations (mean rIQR = 14.2), and the executive, administrative, and managerial occupations (mean rIQR = 13.8); although these groups had on average low median exposures (data not shown). The machine operator, assemblers, and inspectors group had the smallest rIQR (mean rIQR = 1.78) followed by handlers, equipment cleaners, helpers and laborers (mean rIQR = 3.56) and the precision, production, craft, and repair group (mean rIQR = 3.09).

#### 3.2.2. By Occupation

The average rIQR by occupation was 8.3, with a range from 0.08 to 20.4. For high exposed occupations in the original JEM, the highest rIQR was drafting occupations e.g. map makers, blue print engineers, or draftsmen (rIQR = 1.93). By contrast to drafting occupations, the rIQR was nearly 23 times higher than electrician apprentices, the occupation with the lowest rIQR (Table 1). Even though medians for electricians and hoist and winch operators were similar in magnitude to electrician apprentices, their rIQRs were nearly twice as high. Further, some occupations had higher rIQR (cooks: 10 times; janitors and cleaners: 13 times) compared to electrician apprentices, but had lower median exposures overall.

**Table 1:** Select occupations classified as high in the original electric shock JEM.

BOC-90	Occupational Title	Electric Shocks Median	rIQR
576	Electrician apprentices	0.97	0.08
575	Electricians	0.90	0.17
848	Hoist winch operators	0.90	0.14
843	Supervisors, material moving equipment operators	0.39	1.13
436	Cooks	0.35	0.86
653	Sheet metal workers	0.25	1.32
649	Engravers, metal	0.23	1.13
798	Production samplers and weighers	0.22	1.14
825	Railroad brake, signal, and switch operators	0.21	1.14
615	Explosives workers	0.20	1.15
867	Helpers, surveyor	0.20	1.15
448	Supervisors, cleaning and building service workers	0.20	1.4
426	Guards and police, except public service	0.19	1.11
715	Miscellaneous metal, plastic, stone, and glass working machine operators	0.18	1.17
347	Office machine operators, n.e.c.	0.18	1.11
453	Janitors and cleaners	0.17	1.10
368	Weighers, measurers, checkers, and samplers	0.17	1.29
477	Supervisors, farm workers	0.16	1.44
766	Furnace, kiln, and oven operators, except food	0.15	1.13
539	Mechanical controls and valve repairers	0.15	1.27
797	Production testers	0.15	1.33
556	Supervisors, painters, paperhangers, and plasterers	0.15	1.47
217	Drafting occupations	0.14	1.93

Occupations with the highest rIQR previously categorized as medium were: meter readers (rIQR = 11.86), managers, food serving, and lodging establishments (rIQR = 1.86), timber cutting logging occupations (rIQR = 11.84), electrical and electronic repairers (rIQR = 11.83), personal service occupations, not elsewhere classified (rIQR = 11.79), and bus, truck, and stationary engine mechanics (rIQR = 11.78). However, these occupations had high rIQR, because their median exposures were small, effectively 0.01.

### 3.3. Comparison of Original Electric Shock JEM to Expanded JEM

We present a comparison of expert categorization in the original JEM and our expanded JEM. In our prior JEM publication, we reported 103 occupations as highly exposed to electric shocks, whereas in the new JEM, 174 occupations were in the high exposure category using tertile cut points (Table 2). When grouped into low, medium and high using tertile cut points, the agreement between the expanded JEM and original JEM data was 63% (Kappa coefficient = 0.448, *p* = 0). It should be noted, however, that the JEMs are not independent from one another.

**Table 2:** Comparison of the original electric shock JEM and expanded JEM.

Original JEM	Expanded JEM ^1^			
	High	Medium	Low	Total
High	103 (20.6%)	0 (0.0%)	0 (0.0%)	103 (20.5%)
Medium	65 (13.0%)	45 (8.9%)	0 (0.0%)	110 (21.9%)
Low	6 (1.2%)	113 (22.6%)	169 (33.7%)	288 (57.4%)
Total	174 (34.7%)	158 (31.5%)	169 (33.7%)	501

**^1^** Using exposure cut point: High (≥ 67%) and Low (≤33%).

**Figure 3:**
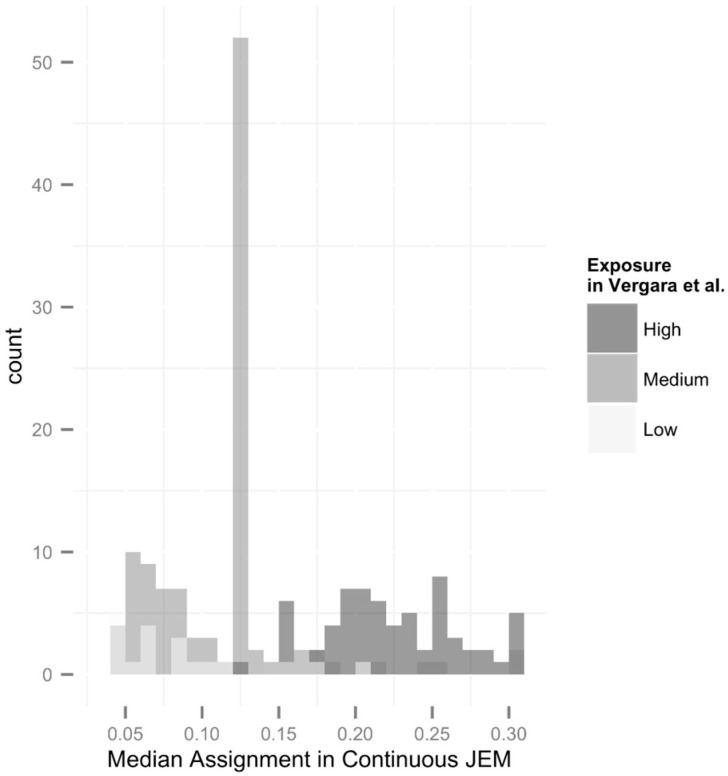
Select range demonstrating changes in distribution between the original JEM and the expanded JEM.

We can compare the median exposures assigned in this new, continuous JEM for those occupations previously given low, medium, and high exposure categorizations (in [5]). The number of overlapping occupations is 200, ranging between the median electric shocks exposures of 0.04 and 0.30 (Figure 3). For example, 22 occupations were given low electric shocks exposure in the original JEM, although their new median exposures ranged between 0.04 and 0.20. Of those, six occupations would be classified as high using the tertile cut points and the remainder as medium electric shocks exposure. In the original JEM, 68 occupations assigned as high electric shocks exposure have median exposure ranges between 0.12 and 0.30, which overlap occupations assigned as medium electric shocks exposure in the original JEM. Six originally assigned low exposure occupations fell into a range of median ranges of 0.12 to 0.20. Approximately, 30 previously assigned high exposure occupations fell into the median ranges of 0.12 to 0.20 in the expanded JEM. Figure 3 plots these overlaps, highlighting differences between the original JEM and the expanded JEM.

We examined changes in electric shocks categorization for select occupations presented in the original JEM paper using tertile and higher cut points (Table 3). For example, pattern makers, layout workers and cutters are classified as a high exposure in the expanded JEM, whereas in the original JEM they were categorized as having medium exposure to electric shocks. A number of the original JEM occupations (103) at high exposure remain so using the expanded JEM with tertile cut points. Most of the original low assignments remain as low, using 75%, cut point with some exceptions, such as sheet metal worker apprentices.

**Table 3:** Electric shock categorical exposure assignments for selected occupations.

BOC-90	Occupation	Electric Shocks					
		Median	Original	Tertiles ^1^	Tertile Binary ^2^ Yes/No	Higher Cut Point ^1^	Higher Cut Point Binary ^2^ Yes/No
575	Electricians	0.90	High	High	1	High	1
526	Household appliance and power tool repairers	0.25	High	High	1	High	1
783	Welders and cutters	0.24	High	High	1	Medium	0
676	Pattern makers, layout workers and cutters	0.12	Medium	High	1	Low	0
538	Office machine repairers	0.13	Medium	High	1	Medium	0
869	Construction laborers	0.38	High	High	1	High	1
436	Cooks	0.35	High	High	1	High	1
804	Truck drivers	0.12	Medium	High	1	Low	0
449	Maids and housemen	0.16	Medium	High	1	Medium	0
666	Dressmakers	0.04	Low	Medium	0	Low	0
744	Textile sewing machine operators	0.03	Low	Medium	0	Low	0
313	Secretaries	0.00	Low	Low	0	Low	0
095	Registered nurses	0.02	Low	Medium	0	Low	0
447	Nursing aides, orderlies, and attendants	0.03	Low	Low	0	Low	0

**^1^** Using electric shocks median, the schemes are as follows: Tertiles: 67% = 0.12, 33% = 0.0004906, Higher cut point: 90% = 0.25, 75% = 0.12; **^2^** Binary exposure: Yes = 1 (High), No = 0 (Medium or Low).

### 3.4. Comparison of Electric Shocks and Magnetic Fields Exposures

Comparing the expanded electric shocks and magnetic fields JEMs as dichotomous variables using high as exposed compared to medium and low as unexposed, the most frequent occupations highly exposed to both electric shocks and magnetic fields were from precision, craft and repair occupations. Common to both exposure schemes, occupations such as textile sewing machine operators and dressmakers were not highly exposed to electric shocks, but were highly exposed to magnetic fields (Table 2).

Upon examination of the expanded electric shocks JEM to magnetic fields, the frequency of occupations differed in discordant cells between the original JEM and its update (Table 4). Unlike the original JEM, 12 occupations were highly exposed to magnetic fields and not to electric shocks (medium or low exposure). Examples of occupations of highly exposed to magnetic fields and not to electric shocks in the expanded JEM included structural metal workers, motion picture projectionists and welders and cutters. Further, 106 occupations were highly exposed to electric shocks and not to magnetic fields unlike the original JEM with 66 occupations. In the original JEM, oil well drillers and machinist apprentices were not exposed to electric shocks or magnetic fields, but in the expanded JEM they are exposed to electric shocks. Some consistency remained between the two electric shocks JEMs, as there were no changes in the occupations classified as highly exposed to electric shocks and not to magnetic fields in the original JEM. When grouped into low, medium and high, the agreement between the expanded JEM and magnetic fields JEM was 40.9% (Kappa coefficient = 0.1061, *p* = 0.0001).

**Table 4:** Exposure contrast frequency of occupation by electric shocks and magnetic fields.

Exposure		Magnetic fields	No magnetic fields	
	Category (Median cut point)	High (≥0.30 µT)	Medium (0.30 µT–0.10 µT)	Low (≤0.1 µT)
Electric shocks	High (≥0.12)	33	90	16
No electric shocks	Medium (0.0004906 > −0.12)	6	113	27
	Low (≤0.0004906)	6	106	26

Electric shocks and magnetic fields exposure cut point schemes noted in parentheses. High exposure contrast cells are shaded. Totals do not equal to 501 as there are no magnetic fields exposure data available for 78 BOC-80 occupational titles.

## 4. Discussion

Job exposure matrices (JEMs) are used to measure exposures based on information about particular jobs and tasks. JEMs are especially useful when individual exposure data cannot be obtained. Information on exposure to electric shocks is limited. We describe a JEM, which defines occupational exposures on a continuous scale and utilizes elicitation methods to quantify exposure uncertainty by assigning exposures probability distributions with parameters determined through expert involvement. The electric shock job exposure matrix was expanded with median proportions, and 25th and 75th percentiles. With these three added dimensions, changes in exposure assignment can be examined more thoroughly prior to epidemiologic analysis and we highlight several examples in this paper.

Overall, occupational electric shocks are rare. High electric shocks exposure on the job is experienced in a few occupational titles. For example, four of 501 occupational titles had at least four in five risk of electric shocks. Further, persons involved directly with electrical work or working in environments near high voltage sources are at highest risk of electric shock. On the other hand, 60 occupational titles had a one in four risk of electric shock on the job, which is low in comparison to higher exposure occupations. Our JEM experts heavily relied on electric injury data (Spearman correlation between elicited medians and data = 0.89) rather than electrocution data (Spearman correlation = 0.39). [9] Due to the rarity of occupational electric shocks, elicitation of small median exposure proportions for each occupation was challenging, similar to other elicitation exercises. [14] The approach used for acquiring such data was iterative, challenging experts to re-examine elicited median proportions against other occupations.

Large rIQR for a given occupation do not indicate high exposure, but instead, demonstrate a need for more information on exposure to electric shocks, whether through improved injury data or expert judgment. Large rIQR can also indicate possible inherent exposure variability within the occupation. For example, for electricians, uncertainty relative to their exposure is small, while for painters, uncertainty relative to their exposure is high.

Whether the elicited median proportion reflects “true” exposure, electric shock for a given occupation remains uncertain. However, given that measurements using classical industrial hygiene methods are not possible, the exposure may only be obtained indirectly, and thus this JEM provides best available information to date on the exposure from electric shocks. Among other strengths of the expanded JEM is that it is population-based; that exposure is continuous making the use of cut-points optional; that it incorporates uncertainty; that it is data driven, but includes expert judgment; and that it allows for a variety of sensitivity analyses.

The choice of cut point strongly affects the number of occupations in the discordant exposure cells (shocks/no magnetic fields or magnetic fields/no shocks). The reduction of false positives in exposure assignment is important for an epidemiologic study. False positives are those occupations that are classified as exposed to electric shocks when they are in fact unexposed. Cut point choice will affect this number, but given the lack of gold standard, the choice of the best cut point is arbitrary. To uncouple the electric shock-magnetic fields relationship, a sufficient part of the study population needs to be exposed to electric shocks and not magnetic fields and *vice-versa*. A continuous exposure based on electric shocks exposure proportions should be used for analysis in concert with ordinal exposure assignments. Sensitivity analysis must be performed to assess how risk estimates from epidemiologic analysis change with revised judgments and cut points, particularly because the assignment to high or medium exposure depends on the cut points used.

Although JEMs are an effective way to assess occupational exposure when data are not available, there are several limitations to consider (as with any form of exposure assessment). As a job is a crude measure of exposure, JEMs lose valuable information at the individual level. Occupational coding schemes are created to capture many occupations, which may vary in tasks, individual worker practices or job conditions and, consequently, impact exposure levels. For example, a non-construction laborer as defined by BOC-90, might encapsulate a resin painter, salvage worker, tool dispatcher or warehouseman, depending on industry. Obviously, electric shock exposure will depend on a variety of factors and might differ substantially between these occupations. Providing measures of uncertainty in the JEM, such as 25th and 75th percentiles, can be used to derive occupation specific exposure distributions and help explain how much information is lacking or how diverse an occupational code might be.

JEMs utilize dimension reduction, which implicitly assumes the absence of any effects of occupational exposure beyond those captured by the JEM. The main purpose of this electric shock JEM is to examine associations between the electric work environment and neurodegenerative diseases, such as ALS. Other occupational exposures, such as welding fumes, may be related to the development of ALS, however, they are unaccounted for when using only the electric shock JEM. The aggregate confounding due to the omitted effects may be important because of the high correlation among various job conditions and worker behavior. These issues can be examined through statistical models, such as mixed models, and other forms of bias analysis. [6,7,15] Improvements in epidemiologic methods will continue to be valuable in the area of occupational epidemiology and statistical analysis of occupational data.

Several avenues of research should be explored: incorporation of severity metric and the addition of industry-specific information. Expert elicitation based on median days away from work involved in non-fatal injury might provide a useful severity metric. Since JEMs need not remain static in nature, newly collected or identified information may be incorporated into them. As highlighted in this paper, information on electric shocks for specific occupations such as drafting, supervisors of farm workers and painters, paperhangers and plasterers or cooks may reduce uncertainties about these exposure proportions. To target specific types of industries, workplace surveys to determine whether persons working in an occupation have had at least one painful work-related electric shock will further reduce uncertainty and fill in the gaps for jobs with non-reportable shocks. The population-based exposure assessment of workplace electric shocks poses significant challenges from the electric shock definition to the capture of less severe events, which are subject to individual worker perception. Research on electric shocks has just begun; it is unclear which type of electric shock, less severe or more severe, is etiologically relevant. Further, low level but frequently occurring shocks might be more etiologically relevant to one particular outcome (e.g. cancer), while infrequent severe shocks might be relevant to other outcomes (e.g. ALS). Nonetheless, advancements in electric shock exposure assessment are possible for reducing uncertainty and knowledge about potential involvement in disease etiology.

Following our initial JEM publication, two new studies on exposure to electric shocks [13,16] were published: an electric shock JEM created for the European population, based on five country registries using 3-digit 1988 International Standard Classification of Occupations (ISCO-88) and a study focused on electrocutions within the construction industry in Taiwan. Both population based electric shocks JEMs consider occupations involved in the installation, repair or maintenance of electrical systems as highly exposed to electric shocks as reported in [13]. Distinct similarities and differences have yet to be explored more formally. Such comparison between our electric shock JEM and the European electric shock JEM, will require manual recoding of occupations from BOC-90 to ISCO-88 codes, but may provide further information on validity and generalizability of electric shock JEMs. Further, these JEMs can be used to evaluate a potential role of electric shocks in a variety of health outcomes provided that the spectrum of exposure, e.g. perceptions to injury, can be refined. Finally, these JEMs identify workers in various workplaces with potential for high electric shocks exposure and thus present opportunities for interventions to prevent death and injury.

## 5. Conclusions 

The changes made to the U.S. general population occupational electric shock JEM produced systematic, consistent exposure estimates. Further, exposure to electric shocks dramatically differs between occupations. Creation and future applications of this expanded JEM will allow for a better understanding of the role of electric shocks in the association between electric occupations and adverse health outcomes, such as ALS reported in previous studies [1].
